# Blunt force trauma and blast injuries to head and chest caused by a potent pyrotechnic device: a case report

**DOI:** 10.1007/s00414-022-02943-6

**Published:** 2023-01-07

**Authors:** Thomas Hunold, Daniel Wittschieber, Christian Ortmann, Senta Niederegger, Niklas Eckardt, Gita Mall, Holger Muggenthaler

**Affiliations:** 1 Institute of Legal Medicine, Jena University Hospital, Am Klinikum 1, 07747 Jena, Germany; 2 Department of Radiology, Jena University Hospital, Am Klinikum 1, 07747 Jena, Germany

**Keywords:** Pyrotechnics, Display shell, Blast lungs, Trauma biomechanics

## Abstract

In times of peace and except for terrorist attacks, fatalities by explosions are rare. Fireworks have deadly potential, especially self-made or illegally acquired devices. The use of professional pyrotechnics by untrained persons poses a life-threatening hazard. We present a case of devastating blunt force and blast injuries to the head and chest of a young man. After ignition of a display shell (syn. a real shell or mortar shell) without the use of a launching pipe, the device hit the man’s face, nearly simultaneously followed by the explosion of the burst charge. The autopsy revealed injuries to the face and forehead as well as extensive tissue structure damage and a massive contusion with a bloody edema of the lungs. Autopsy results are supplemented with CT imaging and 3D reconstruction of the fractured mid face, as well as histological and toxicological examinations. This case of a misused display shell demonstrates both its devastating destructive potential and the corresponding and rarely observed injury pattern.

## Introduction

In times of peace and except for terrorist-attacks, fatalities by explosions are rare [Lit]. Besides some case reports in the forensic literature, German and Austrian newspapers reported on some cases from the New Year’s Eve 2021/22. In this regard, two people were killed by the so-called display shells. A self-made device in Hennef near the city of Bonn (Federal State of North Rhine Westphalia) killed a 37-year-old man (https://www.faz.net/aktuell/gesellschaft/ungluecke/silvester-tote-und-verletzte-bei-feuerwerk-explosionen-17710689.html). A 20-year-old man died in a field near the city of Jena (Federal State of Thuringia) from the impact and explosion of a display shell, and in Berlin, such a device caused injuries in 12 persons (https://www.faz.net/aktuell/gesellschaft/ungluecke/silvester-tote-und-verletzte-bei-feuerwerk-explosionen-17710689.html). In this report, we will present the case of the 20-year-old man from Thuringia.

New Year’s Eve celebrations in Germany and other countries traditionally include privately organized firework displays. Due to coronavirus regulations, the federal government had prohibited the seasonal sale of fireworks to private individuals in 2020 and 2021. This might have led to an increase in purchases of large and illegal fireworks online or in neighboring countries like Poland or the Czech Republic. Such devices may not meet local regulations or require a special usage permit.

**Fig. 1:**
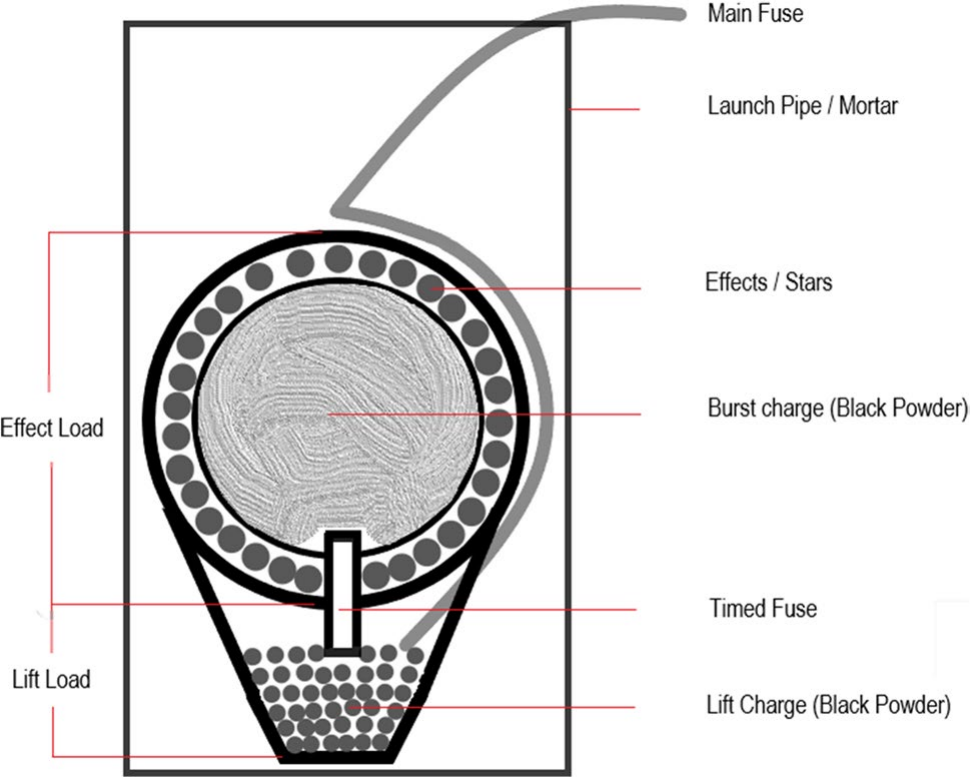
Sketch of a display shell

Display shells are large fireworks classified within safety category F4 (https://www.beuth.de/de/norm/din-en-15947-5/235033731) and suitable only for trained professional pyrotechnicians with usage permits. They are known for spectacular effects like sparkling fireballs in high altitudes and consist of two units (Fig. 1), one for launch and one for detonation. The device needs to be placed in a launch pipe. The attached short main fuse must be extended and is usually provided with an electrical detonator. Ignition of the main fuse must occur from a safe distance. The lift charge then skyrockets the shell while the pipe sets its direction, similar to a mortar. Near the apex of the trajectory, the delay fuse ignites the burst charge, which distributes the exploding effects symmetrically into the sky.

This case impressively shows that improper use of such devices can cause fatal injuries. A literature review places this case in context to similar deaths in the recent past (https://www.faz.net/aktuell/gesellschaft/ungluecke/silvester-tote-und-verletzte-bei-feuerwerk-explosionen-17710689.html, https://www.wienerzeitung.at/nachrichten/chronik/oesterreich/2133014-Mann-in-Baden-durch-Kugelbombe-getoetet.html) [1] (https://www.heute.at/s/todesfalle-kugelbombe-vier-tote-in-10-jahren-100181938). In addition, the pathomechanical basis of the injury pattern is presented and should serve as a reference for similar cases.

## Case report

The police reported on the incident and basics of the display shell construction prior to the forensic autopsy; a 20-year-old man and friends went to a field in order to launch some fireworks. According to witnesses, neither the man nor his companions were under the influence of alcohol or otherwise intoxicated. The police was not able to specify the display shell used. Based on witness statements and after examining the shell fragments, the diameter of the spherical device was assumed to be 2.0–2.5 in or 5–6 cm, and it was probably acquired in Eastern Europe.

Instead of using a launch pipe, the man placed the device on the bare ground. Furthermore, he ignited the main fuse directly rather than attaching an extension as required for such fireworks. Due to the special construction of the main fuse, which has a hidden core of black powder, there are no visible signs of burning, such as sparks or smoke. Witnesses reported that the man bent over the shell attempting to reignite it, probably based on the false assumption that the main fuse was not burning. In this very moment, the lift charge activated and shot the device into the man’s face. The effect-shell shattered on impact, and the burst load exploded. The affected person collapsed, and witnesses described some wheezing as last sign of life in the victim shortly after the incident. The man was pronounced dead in an ambulance after unsuccessful resuscitation efforts.

## Autopsy findings

**Fig. 2:**
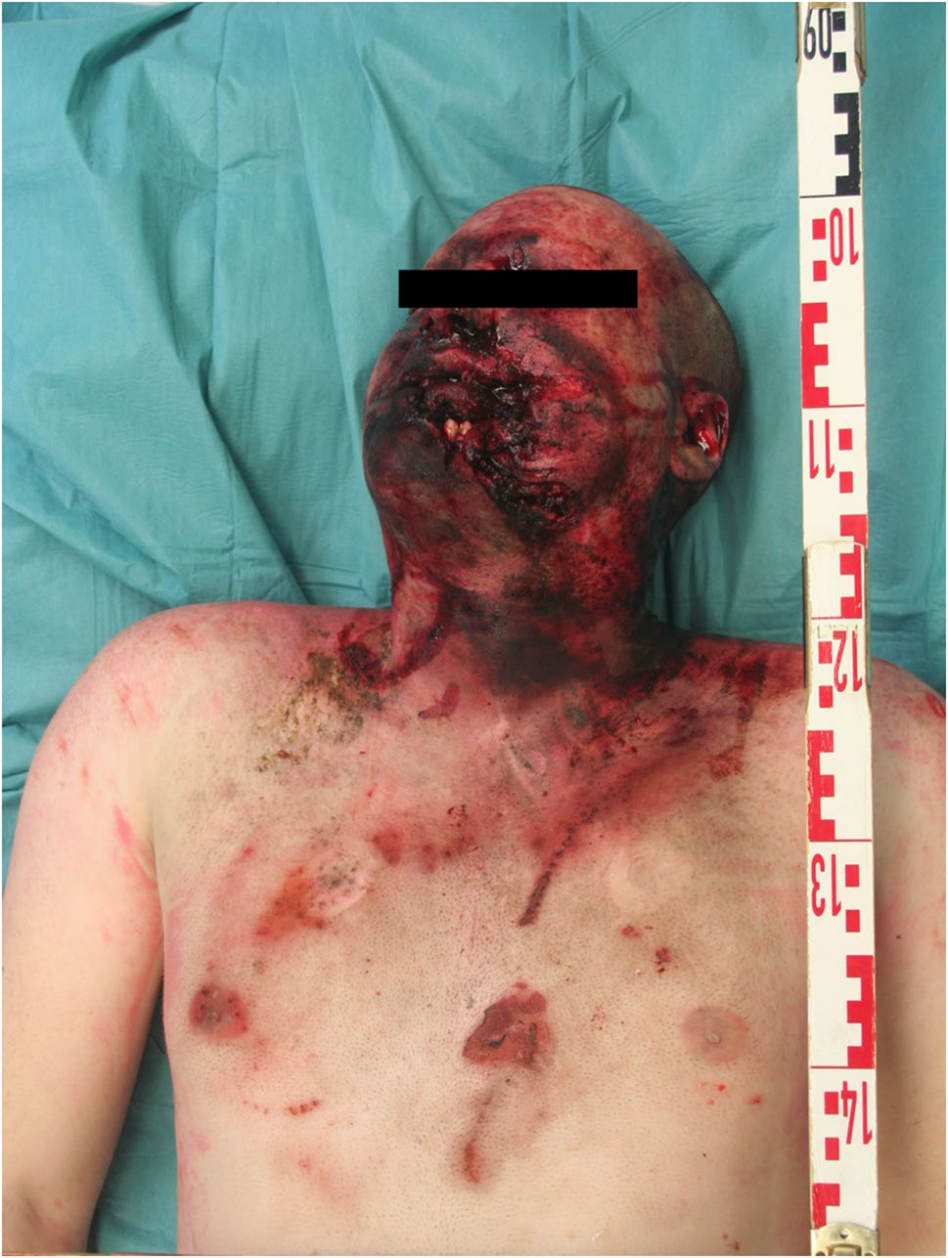
Injuries of the upper body

**Fig. 3:**
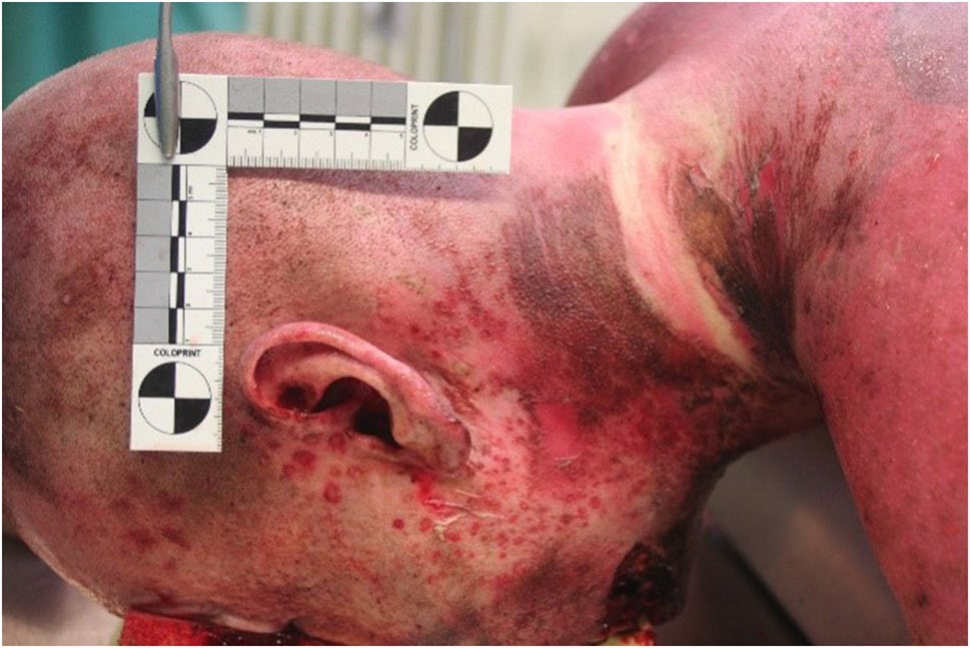
Injuries of the left side of the head and neck

A forensic autopsy was performed about 7 days after death. An external inspection of the corpse showed burn injuries on the face and neck (left side dominant), as well as on the upper chest (Fig. 2) with both reddish skin discoloration and flat black-gray applications and discolorations in accordance with third-degree burns. Furthermore, reddish point-like spots could be associated with scattered sparks and powder residues (Fig. 3).

**Fig. 4:**
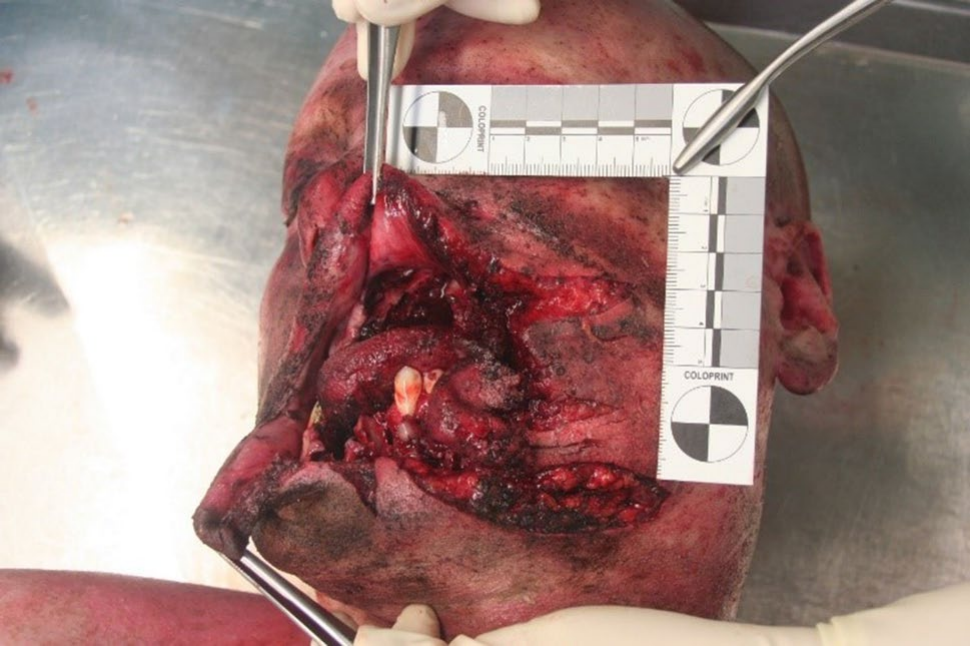
Injuries of the face

**Fig. 5:**
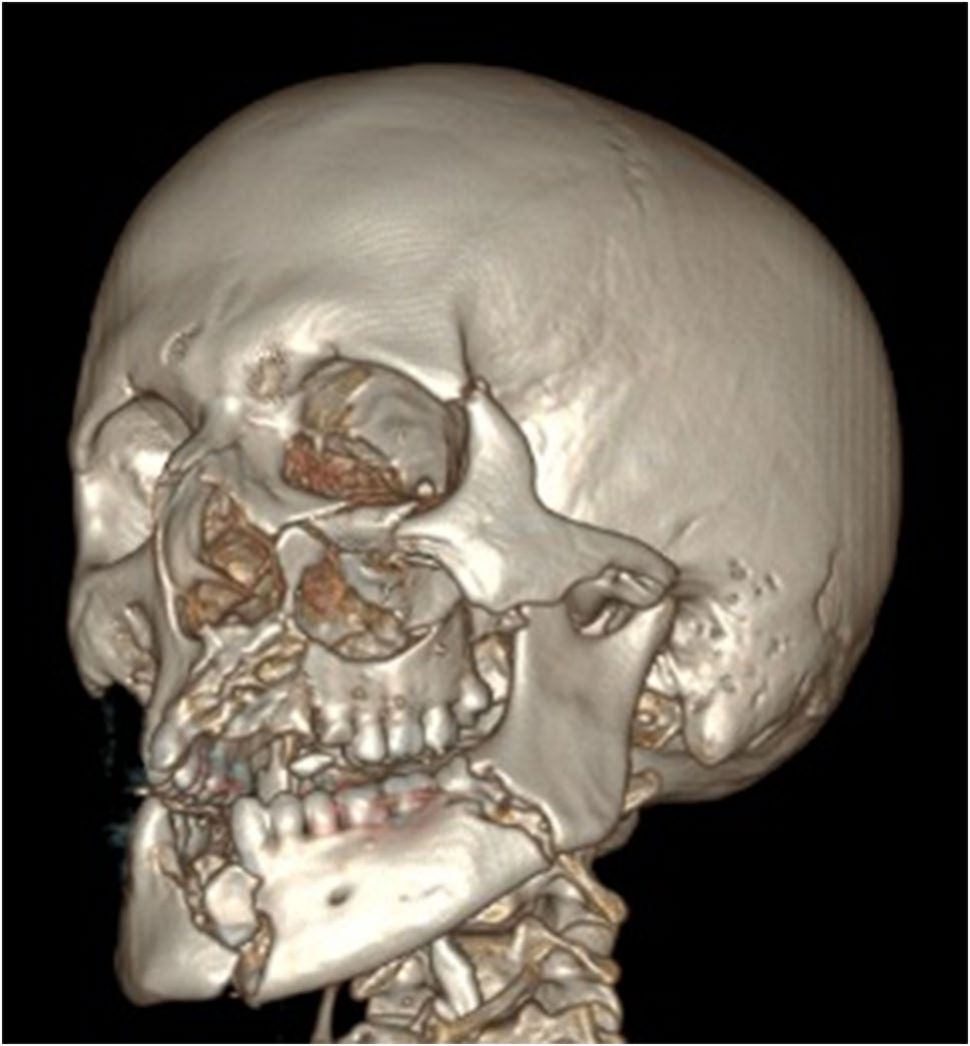
CT imaging–based 3D reconstruction of the mid face and skull

The region comprising the left corner of the mouth and the left cheek appeared as the primary impact site (Fig. 4). This impact led to an irregular laceration of the left facial soft tissue with multiple radial branches. Multiple shreds of soft tissue as well as tooth fragments were found in the center of this area. The preparation of the facial soft tissue and CT imaging confirmed comminuted fractures of the mandible and maxilla as well as the bony eye socket and cheekbone (Fig. 5). In addition, the tongue showed multiple lacerations.

**Fig. 6:**
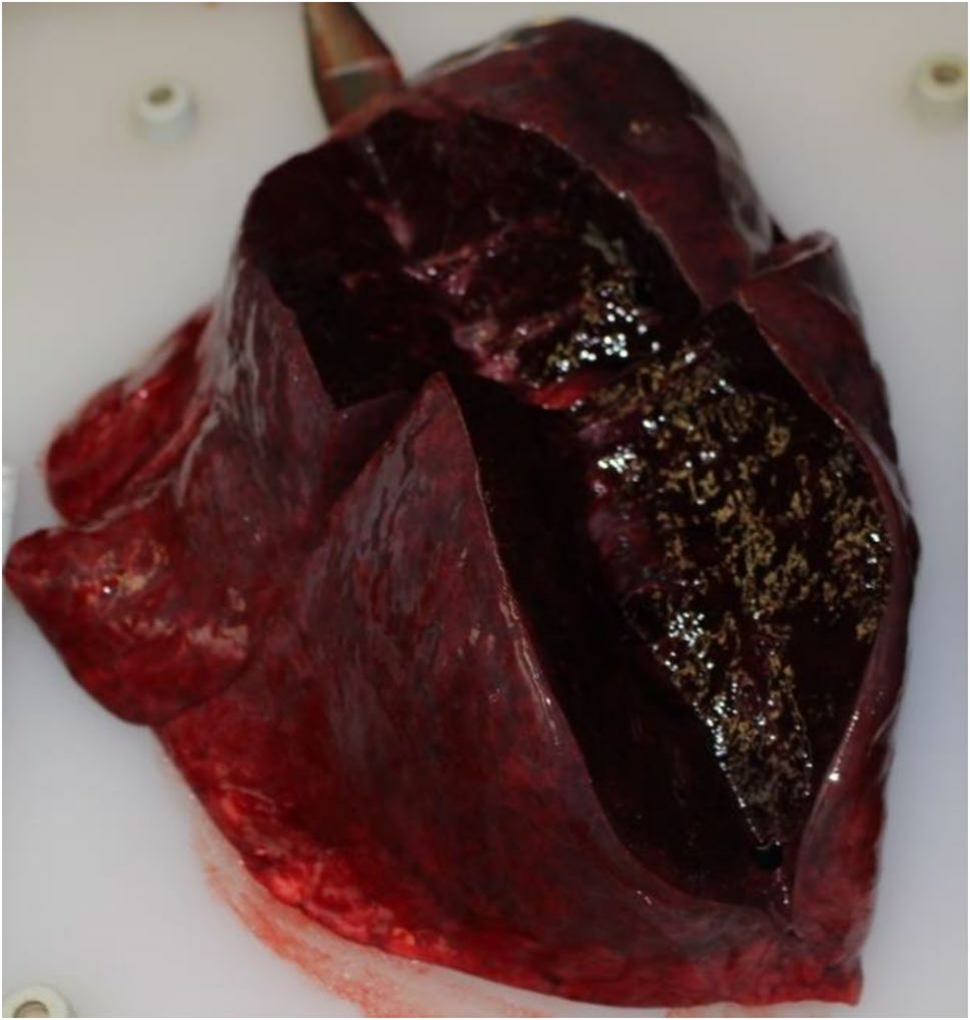
Lung contusion/hemorrhagic lung edema

**Fig. 7:**
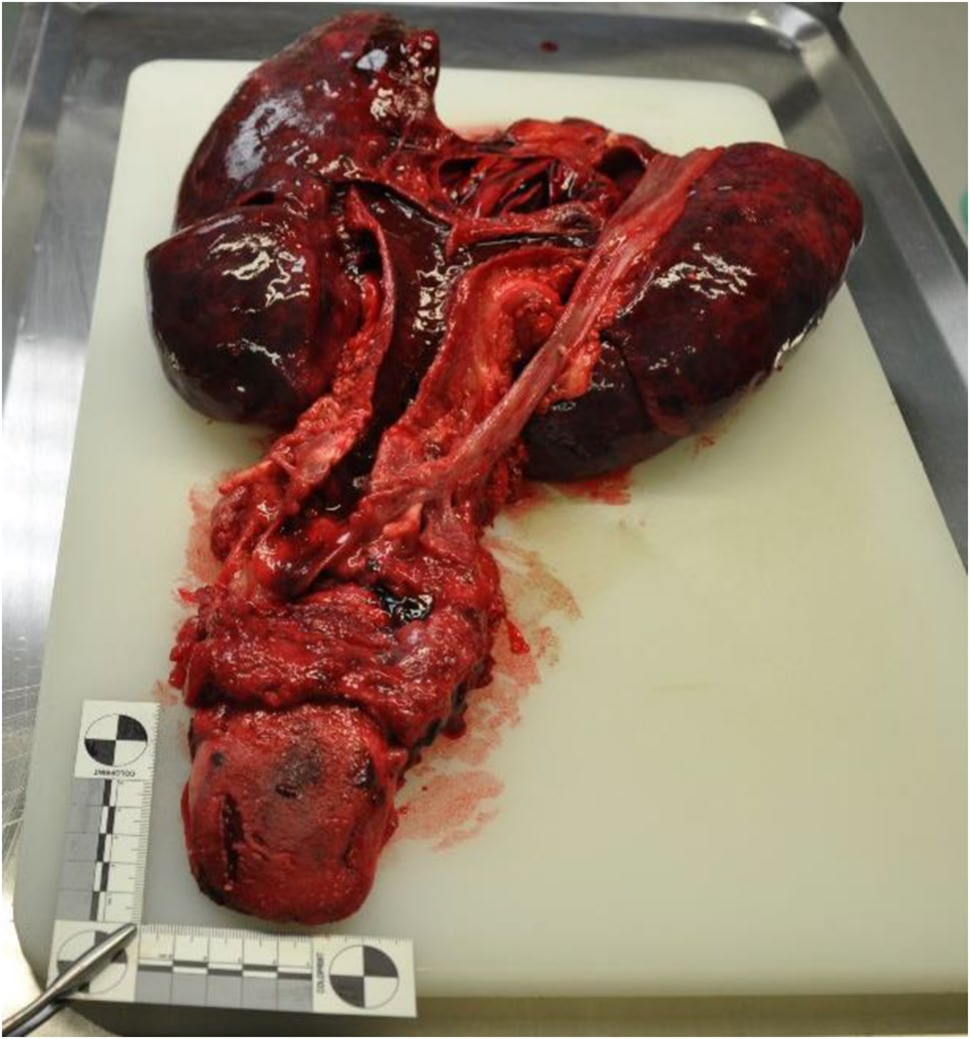
Bloody content in the trachea and lung contusion

**Fig. 8:**
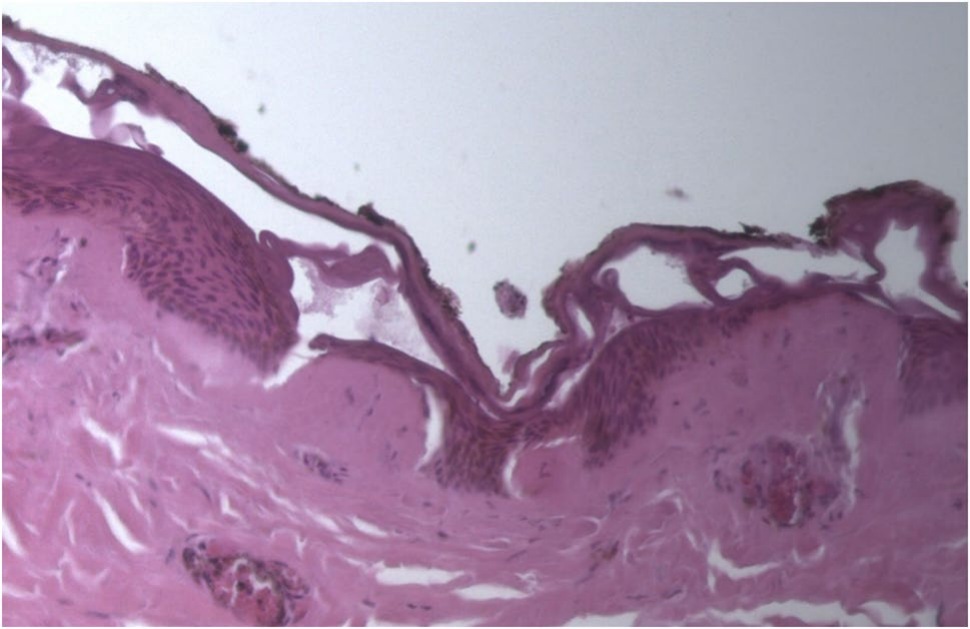
Heat exposure to the skin and burns on left side of the neck

The left hand sustained burn injuries of up to third degree. Neither external nor internal inspection of the skull cavity or the CT imaging revealed any fractures of the skull. Cerebral cortex contusions on the left basal side corresponded to the mid facial trauma as signs of pressure influence. The brain showed an edema and weighed 1642 g. The trachea contained ample hemorrhagic secretions. Both lungs showed relatively high weights (790 g left, 850 g right) and a severe hemorrhagic lung edema (Figs. 6 and 7). The histological investigations of the lung tissue confirmed smashed alveolar septa and bloody contents in the alveoli, as signs of pressure exposure and some chyme in the bronchioles as signs of an agonal aspiration compatible with the last groan described by the witnesses. Remains of powder and smolder were confirmed by microscopy on the mucous membrane of the respiratory tract. Histologically, the burns of the skin showed burned hair residues and epidermal honeycomb-like cavity formations, partly filled with liquid (Fig. 8). The autopsy revealed a cranio-cerebral trauma in combination with a massive lung contusion as the cause of death due to pressure wave. The toxicological analysis showed negative alcohol and drug screening results. The findings correspond with the information on the incident given by the police.

## Discussion

Fatalities in the context of nonprofessional fireworks are rare. However, an internet and literature research revealed some comparable fatalities due to display shell accidents reported in the last years, for example in Germany, Austria (https://www.faz.net/aktuell/gesellschaft/ungluecke/silvester-tote-und-verletzte-bei-feuerwerk-explosionen-17710689.html, https://www.wienerzeitung.at/nachrichten/chronik/oesterreich/2133014-Mann-in-Baden-durch-Kugelbombe-getoetet.html), and the USA [1]. In Austria, four men were killed by display shells in a decade (https://www.heute.at/s/todesfalle-kugelbombe-vier-tote-in-10-jahren-100181938). In addition, intentionally misused display shells may cause injuries in bystanders as in Berlin in 2022 (https://www.berliner-kurier.de/berlin/blutiges-silvester-kugelbomben-attacke-in-friedrichshagen-12-verletzte-li.203576). Besides serious injuries, the explosive power of detonation can also lead to significant property damages [2].

The reports prove the high risk emanating from these pyrotechnic devices with a severity of injuries otherwise only seen in military contexts. “Civilian blast-associated injuries are not common, but they can be severe, and in many (though not all) respects they seem similar to those described in published case series of military blast victims” [3]. Our case confirms this statement. An inquiry of a manufacturer revealed that comparable devices (2–2.5 in) usually contain an estimated amount of explosives of approximately 74 g, mainly black powder. In comparison, a firecracker freely available in Germany has a maximum black powder content of only 6 g (https://www.beuth.de/de/norm/din-en-15947-5/235033731). In the present case, the forensic autopsy including computed tomography, toxicology and histology showed a combination of blast lungs and head injury as the cause of death. The uncommon injury pattern is due to misuse of an illegally acquired display shell, which is provided in Germany for professionals only, and has approximately 12 times the amount of explosives compared to freely available fireworks.

Table 1 summarizes different blast injury mechanisms categorized as primary, secondary, tertiary, or quaternary injuries. In our case, we found primary injuries in terms of blast lungs and secondary injuries in terms of blunt force trauma.

**Table 1:** Categories of blast injuries (from (https://web.archive.org/web/20080913225423/http://www.bt.cdc.gov/masscasualties/blastessentials.asp))

Primary injury	Injury from over-pressurization force (blast wave) impacting the body surface Tympanum rupture, pulmonary damage and air embolization, hollow viscus injury
Secondary injury	Injuries from projectiles (bomb fragments, flying debris) Penetrating trauma, fragmentation injuries, blunt trauma
Tertiary injury	Injuries from displacement of victim by the blast wind Blunt/penetrating trauma, fractures and traumatic amputations
Quaternary injury	All other injuries from the blast Crush injuries, burns, asphyxia, toxic exposures, exacerbations of chronic illness

According to Karger [4], blast lungs as well as air embolisms are the major causes of death in such cases. In our case, air embolism was found in the CT imaging within the coronary arteries and in the liver. However, this could be attributed to the long interval between death and autopsy due to putrefaction, so blast lung-associated air embolism cannot be proved. Blast lung injuries were described by Karger [4] to be a result of blast-wave exposure and appear as heavy and stiff lung tissue with subpleural contusions. The lungs in our case appeared as described above. The complete parenchymal tissue was hemorrhagic, which can be interpreted as a result of the close proximity of the detonation and the power of the detonation wave to the chest. We observed a displacement of teeth and tissue into the upper respiratory tract as well as tracheal mucous burns and remains from powder and soot as signs of exposure to heat and pressure by explosion. Presumably, this is due to the proximity of the explosion to the oral cavity. The massive brain edema and basal contusions can be ascribed to the impact of the blast wave. “Hyperemia and severe cerebral edema occur frequently in patients who sustain significant blast traumatic brain injury” [5]. The head injury with trauma to the mandible and maxilla and fractures of the bony eyesocket are caused by the direct impact of the device (secondary category of blast injuries, Table 1).

The severity of injuries concerning the lungs and the head with extensive lacerations of the facial soft tissue indicate high pressure and blunt force by direct impact. According to literature, legal fireworks can cause injuries of the head [6, 7], particularly involving the eyes and ears. Serious injuries mainly affect the upper extremities [7, 8]. In a survey (https://www.presseportal.de/pm/156523/5009947#) by a German manufacturer, 1800 participants were asked about injuries caused by fireworks in the last 10 years, and 72% (of 269 injured individuals) have indicated that they have suffered injuries to one hand, 14.6% involving the ears, 11.4% the eyes, and 12% the face (without eye injuries).

The literature and public media (https://www.kleinezeitung.at/lebensart/gesundheit/5344904/Aerzte-warnen_Alkohol-und-Feuerwerk_So-gefaehrlich-ist-diese-Kombi) typically report on firework accidents in the context of alcohol consumption. On the other hand, a Danish study [9] showed that only one of eight decedents in firework-related deaths was intoxicated with alcohol. In our case, neither alcohol nor other substance misuse could be detected.

Covid regulations prohibiting public firework in Germany in 2021/2022 resulted in a nationwide decrease of 40% in hospitalizations due to injuries caused by fireworks [10]. In contrast, more injuries were reported during the same time in the USA due to an increase in consumer firework sales [11]. Regulations will neither prevent illegal acquisition of fireworks nor prevent misuse by untrained individuals. Provision of information about the dangers that emanate from pyrotechnic devices, however, is more important than ever.

## Data Availability

Not applicable.
